# The Actin Gene Family: Function Follows Isoform

**DOI:** 10.1002/cm.20475

**Published:** 2010-08-24

**Authors:** Benjamin J Perrin, James M Ervasti

**Affiliations:** Department of Biochemistry, Molecular Biology and Biophysics, University of MinnesotaMinneapolis, Minnesota

## Abstract

Although actin is often thought of as a single protein, in mammals it actually consists of six different isoforms encoded by separate genes. Each isoform is remarkably similar to every other isoform, with only slight variations in amino acid sequence. Nevertheless, recent work indicates that actin isoforms carry out unique cellular functions. Here, we review evidence drawn from localization studies, mouse models, and biochemical characterization to suggest a model for how in vivo mixing of actin isoforms may influence cytoskeletal function in cells. © 2010 Wiley-Liss, Inc.

## Introduction

**Fig. 1:**
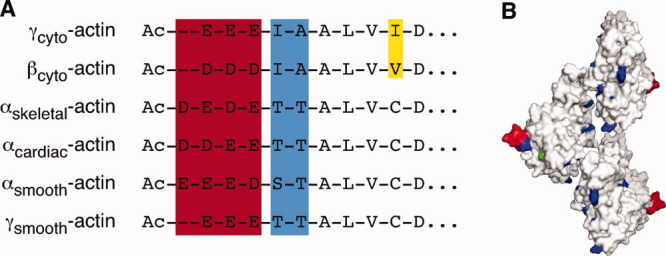
Actin-isoform sequence differences **A**: Alignment of the N-terminal ends of the six mammalian actin isoforms. **B**: Amino acid differences mapped onto F-actin structure. The residues in red exhibit the most variability within and between muscle and cytoplasmic isoforms. Blue residues primarily vary between cytoplasmic and muscle isoforms. Yellow varies between β_cyto_-actin and γ_cyto_-actin while green indicates substitutions between different muscle isoforms. The F-actin structure is approximate and based on the work of Oda et al. [PDB 2ZWH, Oda et al., 2009].

Actin is essential for a tremendous range of cell functions. A partial list includes cell division, migration, junction formation, chromatin remodeling, transcriptional regulation, vesicle trafficking, and cell shape regulation. How is it possible that one molecule can accomplish such a large diversity of tasks? One answer to this mystery is that actin is not a single entity; rather, actin is composed of several different isoforms. Birds and mammals have six genes, and each encodes one protein isoform. Four isoforms, α_skeletal_-actin, α_cardiac_-actin, α_smooth_-actin, and γ_smooth_-actin, are expressed primarily in skeletal, cardiac, and smooth muscle. The remaining two isoforms, β_cyto_-actin and γ_cyto_-actin are ubiquitously expressed. All of the isoforms possess very similar amino acid sequences, with no isoform sharing less than 93% identity with any other isoform. β_cyto_-Actin and γ_cyto_-actin, which are exactly conserved from birds to mammals, only differ by four biochemically similar residues (Fig. 1). In an unknown fashion, these family members cooperate to endow the microfilament network with diverse properties. Here, we will describe evidence from mouse models supporting the notion that actin isoforms have specialized cellular functions and discuss possible ways in which actins could exert differential effects in cells.

## Experimental Evidence for Actin-Isoform Specific Functions

Studies in model organisms including *Drosophila* [Wagner et al., 2002] and *C. elegans* [MacQueen et al., 2005] have provided a rich source of evidence to suggest that actin isoforms have both overlapping and unique cellular functions. In mammalian systems, most evidence has come from mouse models where individual actin-isoform knockouts have distinct phenotypes. In addition, transgenic actin expression studies have demonstrated some overlap and some special properties among actin isoforms.

### Muscle Isoforms

All knockout mice reported thus far have distinct phenotypes suggesting that each isoactin has unique and specialized functions (Table I). α_cardiac_-Actin knockout results in lethality during either embryonic or perinatal development with profound disorganization of cardiac myofibrils [Kumar et al., 1997]. α_skeletal_-Actin knockout mice appear normal at birth but have weak muscles and all die by 9 days of age [Crawford et al., 2002]. α_smooth_-Actin knockout mice are born at Mendelian ratios and are viable. However, these mice have defects in vascular contractility and blood pressure regulation [Schildmeyer et al., 2000]. γ_smooth_-Actin knockout mice have not yet been reported.

**Table I:** Actin-Isoform Mouse Models

Protein ablated	Gene	Allele	Transgenic rescue	Phenotype	References
α_skeletal_-Actin	*Acta1*	Null		Pups die by 9 days of age; exhibit muscle weakness	Crawford et al., 2002
			α_cardiac_-Actin	Full rescue	Nowak et al., 2009
			γ_cyto_-Actin	Does not rescue	Jaeger et al., 2009
α_cardiac_-Actin	*Actc1*	Null		Embryonic/perinatal death; disorganized myofibrils	Kumar et al., 1997
α_cardiac_-Actin			γ_smooth_-Actin	Partial rescue of lethality; hearts defective	Kumar et al., 1997
α_smooth_-Actin	*Acta2*	Null		Viable; defects in vascular contractility and blood pressure regulation	Schildmeyer et al., 2000
β_cyto_-Actin	*Actb*	Hypomorph		Embryonic lethal	Shawlot et al., 1998; Shmerling et al., 2005
γ_cyto_-Actin	*Actg1*	Null		Reduced viability; small size; progressive deafness	Belyantseva et al., 2009; Bunnell and Ervasti, 2010
γ_cyto_-Actin		Conditional-skeletal muscle		Progressive centronuclear myopathy	Sonnemann et al., 2006

In all of these mouse models, there is compensatory upregulation of a subset of the remaining actin isoforms. However, since each muscle actin is highly expressed the total level of actin in knockout muscle cells remains less than in wild type. Therefore, transgenic overexpression of other isoforms may rescue the knockout phenotype and provide a useful measure of overlapping function between the isoforms. Along these lines, γ_smooth_-actin was overexpressed in α_cardiac_-actin knockouts [Kumar et al., 1997] and α_cardiac_-actin was overexpressed in α_skeletal_-actin knockouts [Nowak et al., 2009]. Overexpression of γ_smooth_-actin only partially rescued mice lacking α_cardiac_-actin. Even when these mice survived to adulthood, their hearts were hypodynamic and hypertrophic, demonstrating that γ_smooth_-actin and α_cardiac_-actin each make distinct contributions to cardiac cell function [Kumar et al., 1997].

α_cardiac_-Actin and α_skeletal_-actin are 99% identical and have overlapping expression patterns [Vandekerckhove et al., 1986]. These similarities suggest that these isoforms have considerable overlapping functions. Correspondingly, transgenic expression of α_cardiac_-actin fully rescued the lethality and muscle performance deficits associated with the loss of α_skeletal_-actin suggesting a potential treatment for human diseases associated with reduced α_skeletal_-actin [Nowak et al., 2009]. In contrast, overexpression of γ_cyto_-actin did not rescue lethality due to α_skeletal_-actin deficiency suggesting that muscle and nonmuscle actins are more specialized. Interestingly, transgenic expression of γ_cyto_-actin in wild-type mice resulted in the substitution of 40% of thin filament α_skeletal_-actin with γ_cyto_-actin [Jaeger et al., 2009]. Therefore, muscle cell function depends on muscle-specific actin isoforms, but can tolerate a surprising amount of contamination by γ_cyto_-actin.

### Cytoplasmic Isoforms

β_cyto_-Actin and γ_cyto_-actin are nearly identical proteins that differ by only four biochemically similar amino acids, all of which are found in the 10 N-terminal residues (Fig. 1A). Mice homozygous for hypomorphic alleles of *Actb* die during early development of uncharacterized defects [Shawlot et al., 1998; Shmerling et al., 2005]. A conditional knockout has been generated, but not yet described (our unpublished data). In contrast to β_cyto_-actin-deficient mice, whole-body γ_cyto_-actin knockout mice are viable and can survive to adulthood and beyond [Belyantseva et al., 2009; Bunnell and Ervasti, 2010]. Interestingly, these mice are smaller than wild-type or heterozygous littermates from an early developmental stage, but are born at normal Mendelian ratios. γ_cyto_-Actin confers a clear survival advantage as significant numbers of newborn *Actg1^−/−^* mice die as a result of developmental delays and another fraction die stochastically during adulthood [Belyantseva et al., 2009; Bunnell and Ervasti, 2010]. Corresponding to decreased animal survival, primary γ_cyto_-actin-deficient fibroblasts cultured from these mice have decreased viability, impaired growth, and increased apoptosis and necrosis. On the other hand, consistent with the viability of *Actg1^−/−^* mice, γ_cyto_-actin null fibroblasts have normal migration in a scratch wound assay [Bunnell and Ervasti, 2010].

Loss of γ_cyto_-actin has similar consequences for cells in vivo. Sensory hair cells in the inner ear depend on stereocilia formed of β_cyto_-actin and γ_cyto_-actin for proper function. γ_cyto_-Actin-deficient hair cells develop normally but do not maintain stereocilia during aging, which corresponds to progressive hearing loss and deafness [Belyantseva et al., 2009]. Similarly, conditional knockout of γ_cyto_-actin in muscle did not have developmental consequences [Sonnemann et al., 2006] despite in vitro evidence that γ_cyto_-actin is required for sarcomere assembly [Lloyd et al., 2004]. Instead, mice developed a novel progressive centronuclear myopathy. In all cell types from γ_cyto_-actin knockout mice studied thus far, the total cellular concentration of actin remains constant due to compensatory upregulation of the other actin isoforms [Belyantseva et al., 2009; Bunnell and Ervasti, 2010]. Since the actin concentration remains constant, the observed phenotypes can be ascribed to changes in the actin composition.

## Mechanisms of Isoform-Specific Functions

There are two commonly held ideas to explain how different isoactins might perform distinct cellular functions. First, a subset of actin-binding proteins could bind specifically to a single isoform and require that interaction for its function. Consistent with this idea, several proteins, including cofilin [De La Cruz, 2005], ezrin [Yao et al., 1996], l-plastin [Namba et al., 1992], βCAP73 [Shuster et al., 1996], Thymosin b4 [Weber et al., 1992], and profilin [Larsson and Lindberg, 1988] have been described that discriminate between muscle and cytoplasmic actin isoforms. In addition, annexin 5a may preferentially bind to γ_cyto_-actin over β_cyto_-actin [Tzima et al., 2000; Wagner et al., 2002]. The second idea is that actin isoforms are localized to distinct subcellular regions, perhaps as a result of differential interactions with actin-binding proteins or by a mechanism that targets transcripts. Differential localization of actin isoforms has been observed in a variety of cell types, with some discordance between reports as to the precise localization patterns for different actins.

### Distinct Localization Patterns

Skeletal muscle provides a seemingly clear example of differential localization of muscle and nonmuscle actin isoforms with α_skeletal_-actin being confined to the sacromeric thin filaments. In contrast, γ_cyto_-actin, which has the best-described localization pattern in muscle, appears to be absent from thin filaments, but is instead found in other muscle cell structures. γ_cyto_-Actin was initially detected in filamentous structures surrounding mitochondria and adjacent to the sarcolemma [Craig and Pardo, 1983; Pardo et al., 1983]. Subsequent reports found that γ_cyto_-actin is the only actin species detected at costameres [Rybakova et al., 2000], which are structures found between the sarcolemma and the z-disk [reviewed in Ervasti, 2003]. Other recent studies detected γ_cyto_-actin in costameres as well as in a novel zone adjacent to the z-disk [Kee et al., 2004]. Finally, different groups have detected γ_cyto_-actin only in z-disks and not in costameres [Nakata et al., 2001; Papponen et al., 2009].

In other cell types, γ_cyto_-actin seems to be uniformly distributed in all actin-containing structures [Otey et al., 1986]. In contrast, β_cyto_-actin seems to have a more polarized distribution [Hoock et al., 1991; Bassell et al., 1998]. The differences between β_cyto_-actin and γ_cyto_-actin localization may be due to a 54-nucleotide ÓzipcodeÓ sequence found in the 3′ UTR of β_cyto_-actin but not γ_cyto_-actin transcripts. The zipcode sequence binds to zipcode-binding protein (ZBP1, also known as IMP-1), which facilitates targeting of the transcript and regulates translation [reviewed in Condeelis and Singer, 2005]. ZPB1 binds numerous different transcripts [Jonson et al., 2007], perhaps integrating β_cyto_-actin into a broader cellular program.

ZBP1-mediated regulation of β_cyto_-actin has important functional consequences in different cell types. In neurons cultured from *X. laevis*, growth cones exposed to an attractive cue require targeted β_cyto_-actin transcripts and newly synthesized β_cyto_-actin protein for normal turning behavior [Leung et al., 2006; Yao et al., 2006]. β_cyto_-Actin is enriched compared to γ_cyto_-actin on the side of the growth cone exposed to attractant [Yao et al., 2006] and growth cones fail to turn when neurons are treated with antisense oligos to β_cyto_-actin [Leung et al., 2006]. Together, this evidence strongly predicts that β_cyto_-actin is essential for normal neuronal development. However, this hypothesis has yet to be tested in an intact mammalian system.

Zbp1-mediated targeting of β_cyto_-actin transcripts appears to be important in other cell types, including fibroblasts and adenocarcinoma cells, because interference with the ZBP1 activity alters cell morphology and migration [Kislauskis et al., 1994; Shestakova et al., 2001]. Consistent with targeting of β_cyto_-actin transcripts, several groups report that β_cyto_-actin is enriched at the leading edge of cultured fibroblasts and myoblasts as compared to stress fibers found in the central region of the cell [Hoock et al., 1991; Hill and Gunning, 1993; Kislauskis et al., 1997; Shestakova et al., 2001]. In contrast, γ_cyto_-actin appears to be uniformly distributed in all actin-containing structures in fibroblasts [Otey et al., 1986].

In a conflicting report, recent work using newly generated isoform-specific antibodies and a different fixation technique suggests that β_cyto_-actin predominates in stress fibers while γ_cyto_-actin is enriched at the leading edge [Dugina et al., 2009]. Discrepancies in β_cyto_ and γ_cyto_-actin localization have also been noted in auditory hair cell stereocilia. Here, immunofluorescent labeling indicated that γ_cyto_-actin was localized at the periphery of stereocilia [Belyantseva et al., 2009], while a study using immuno-TEM found that β_cyto_-actin was peripheral and γ_cyto_-actin was more centrally localized [Furness et al., 2005].

Study-to-study differences in observations of cytoplasmic actin localization in cultured cells, stereocilia, and muscle indicate that actin localization presents a formidable challenge. The slight amino acid sequence differences between isoforms require establishing antibody specificity, ideally using knockout tissue. Importantly, antibody binding interactions vary with experimental conditions; for example, the specificity of the anti-a_cardiac_-actin antibody depends on the salt concentration [Franke et al., 1996]. Even with validated antibodies, it is difficult to interpret cases where there is a lack of staining (i.e., costameres, the central regions of stereocilia, stress fibers) because it seems that some actin-based structures may be inherently difficult to immunostain. Tightly bundled actin filaments, such as those found in stereocilia and stress fibers, may resist staining due to epitope masking or limited antibody penetration. Other actin-based structures, such as costameres, are labile and easily lost during fixation.

### Actin as an Alloy

Differential localization as a mechanism to explain the distinct functions of actin isoforms is an attractive hypothesis because of its intuitive simplicity. However, it remains unclear how relative enrichment leads to changes in actin function. New biochemical evidence suggests that F-actin properties may vary according to the mix of isoforms in the filament. Bergeron et al. [ 2010] recently characterized purified β_cyto_ and γ_cyto_-actin produced in a recombinant baculovirus system. They observed that under calcium-bound conditions, β_cyto_-actin exhibited more dynamic behavior than γ_cyto_-actin with faster polymerization and depolymerization rates. Intriguingly, biochemical assays demonstrated that β_cyto_ and γ_cyto_-actin readily copolymerize and that the resulting filaments have polymerization and depolymerization rates that vary according to the ratio of β_cyto_-actin to γ_cyto_-actin [Bergeron et al., 2010]. In addition to polymerization dynamics, different mixtures of actin isoforms may well have distinct biophysical properties or distinct interactions with stability regulators such as AIP1 or cofilin.

Varying the mixture of actin may be a useful mechanism for adapting the cytoskeleton for a variety of functions. By way of analogy, steel is an alloy composed of numerous different metals. The ratio of each component determines the characteristics of the particular kind of steel, which is engineered to meet the desired compromises between properties such as weight, tensile strength, and cost. It is important that alloys can be manipulated because innumerable different steels are required to meet the varied demands of buildings and machines. Actin is cellular steel. Altering the ratio of actin isoforms within a filament may tune its properties to meet the specific requirements of different cells or subcellular structures.
